# Acute leukaemia in children with Down syndrome in a low middle-income country

**DOI:** 10.3332/ecancer.2022.1374

**Published:** 2022-04-13

**Authors:** Rahat Ul-Ain, Mahwish Faizan, Saadia Anwar, Shazia Riaz, Alia Ahmad, Huma Zafar, Wasila Shamim

**Affiliations:** Department of Pediatric Hematology/Oncology & Bone Marrow Transplant, University of Child Health Sciences, Children’s Hospital, Lahore, Punjab 54600, Pakistan; a https://orcid.org/0000-0001-8492-1293

**Keywords:** Down syndrome, acute leukaemia, childhood leukaemia, low middle-income country

## Abstract

Down syndrome (DS) is the commonest chromosomal disorder and is considered to be the most common syndrome associated with acute leukaemia. The objective of this study was to determine the characteristics of acute leukaemia in children with DS in Pakistan. It was a retrospective, cohort study conducted over a 2-year period, and the data was analysed in SPSS 20.0 in terms of descriptive statistics. Nineteen DS patients with acute leukaemia were enrolled. The proportion of DS-acute leukaemia was found to be 1.84% among all cases of paediatric acute leukaemia. The mean age of presentation was 5.5 years ± 4.3 SD with a male to female ratio of 1.1:1. The precursor B-cell ALL was found in 13 (68.4%) and acute myeloid leukaemia was found in 6 (31.6%) patients of DS. Thirteen patients (68.4%) completed treatment, while 6 (31.6%) expired due to treatment-related toxicity. Mean overall survival was 38 months ± 5.34 SD. The status of diagnosis of DS before presentation with acute leukaemia was the only statistically significant factor associated with the outcome. Few distinct characteristics of DS-acute leukaemia have been found in our population. Treatment toxicity was the sole cause of treatment failure.

## Introduction

Down syndrome is the most common chromosomal disorder with an incidence of 1 in 700 births in the United States. It is associated with an increased risk for certain medical conditions including childhood leukaemia [1]. There is a 10–20-fold increased risk of developing acute leukaemia in children with DS, with a cumulative risk of 2.1% at the age of 5 years [2, 3]. Leukaemia is one of the most common causes of mortality in patients with DS [3]. The prognosis of Down syndrome-acute lymphoblastic leukaemia (DS-ALL) has been considered poorer as compared to non-DS-ALL [5–7], but the recent trials have shown excellent long-term survival of children with DS-ALL and increased rate of toxicity [8, 9]. Down syndrome-acute myeloid leukaemia (DS-AML), on the other hand, is known for its favourable outcome [10–15]. In the past few decades, the life expectancy of DS has dramatically increased in the developed world [1]. Developing countries, like Pakistan, are yet far away from these goals. The most important milestone lacking, in this regard, is the basic statistics of the disease and research. There are limited data available from developing countries regarding DS with childhood acute leukaemia.

The objective of this study was to determine the frequency of DS-associated paediatric leukaemia among all paediatric leukaemia cases, including the outcome, factors associated with outcome and the causes of mortality in patients with DS with paediatric acute leukaemia in our country.

## Materials and methods

This was an observational; retrospective, cohort study conducted in the Department of Paediatric Haematology/Oncology at the Children’s Hospital, Lahore, Pakistan. Approval from the institutional ethical committee was taken. The data was collected retrospectively over a 2-year period, i.e., from January 2017 to December 2018. All children of DS, aged more than 1 year and less than 16 years, newly diagnosed with acute leukaemia, from January 2017 to December 2018, were included in the study. Patients not having DS, aged less than 1 year and/or with transient abnormal myelopoiesis/transient myeloproliferative disorder were excluded from the study. The data was collected retrospectively from ward records and the study cohort was followed up till July 2021 regarding the outcome of the cohort in terms of disease-free survival/overall survival. The data were analysed in terms of descriptive statistics with SPSS version 20.0. The range of the observation time was 31–54 months after diagnosis and the mean time of observation was 42.5 months after diagnosis. Chi-square test was applied for the determination of *p*-values, and the survival outcome was analysed with the Kaplan–Meier method and the log-rank test.

The Paediatric Haematology/Oncology Unit at the Children’s Hospital, Lahore, is Pakistan’s largest, public sector, 100-bedded subspecialty unit. It offers treatment and palliative care, free of cost, to paediatric patients with all kinds of malignant disorders, from all over the country, as well as from the neighbouring country, Afghanistan.

DS-ALL patients were treated as per UKALL2011 trial [16], while DS-AML patients were treated as per COG A2971 [17] protocol. Supportive care was given to all patients as per the treatment protocols. Tumour lysis syndrome prophylaxis was given to all patients on admission and pneumocystis jiroveci prophylaxis (trimethoprim–sulfamethoxazole) was given to all patients throughout the treatment. Antibiotic prophylaxis with fluoroquinolones was not given to any patient.

## Operational definitions

***Down syndrome:*** Patients with DS were diagnosed based on clinical features and/or karyotype suggestive of DS.

***Acute leukaemia:*** Patients with acute leukaemia were diagnosed based on flow cytometry with immunophenotype suggestive of various types of acute leukaemia.

***Sepsis:*** Sepsis was defined as clinical or laboratory evidence of infection with systemic inflammatory response syndrome.

***Invasive Fungal Infection:*** It was diagnosed based on the presence of neutropenia or any other immunocompromised state, along with sufficient clinical evidence consistent with invasive fungal disease (without mycological support).

## Results

**Figure 1.**
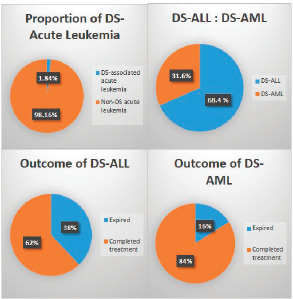
Proportion, types, and outcome of paediatric acute leukaemia with Down syndrome.

**Figure 2.**
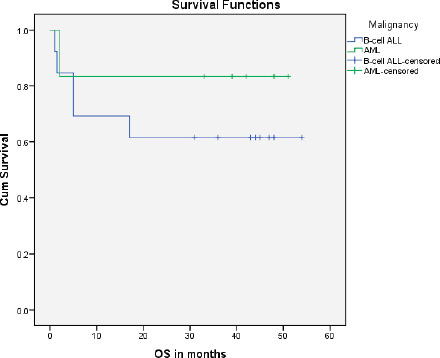
Survival curve for DS-AL patients.

A total of 1,035 paediatric patients with acute leukaemia were registered over the 2-year study period (871 with ALL and 164 with AML). Nineteen (1.84%) patients were found to have DS and therefore included in the study (Figure 1). The median age of presentation of DS-acute leukaemia was 5.5 years ± 4.3 SD, with an age range of 14.00 years, and a male to female ratio of 1.1:1. DS-associated precursor B-cell ALL (B-ALL) had a proportion of 1.5% among all paediatric ALL cases while DS-AML cases were 3.7% of all paediatric AML cases. None of the patients with DS presented with T-cell ALL or mature B-cell immunophenotype. The median age at diagnosis of DS-ALL was 4 years, while that of DS-AML was 6 years. Twelve patients (63%) were already diagnosed as DS and were on regular follow-up with their primary physicians, while the remaining seven patients (37%) were diagnosed with DS at the time of presentation with acute leukaemia. Regarding the outcome of the study cohort, 13 patients (68.4%) completed treatment successfully and are alive, while 6 patients (31.6%) expired during treatment (Figure 1). The mean overall survival of all of the DS patients with acute leukaemia was 38.6 months ± 5.25 SD. The mean overall survival in children with DS-ALL was 35.5 months and 42.8 months in DS-AML (Figure 2). The log-rank test showed that there was no statistically significant difference in the overall survival distributions between the two groups, i.e., DS-ALL and DS-AML (*p*-value = 0.388).

Regarding the cohort of patients who expired, five patients (83%) had B-ALL and one patient (17%) had AML (38% of DS-B-ALL and 16% of DS-AML expired). All the patients had treatment-related mortality and none of the patients expired due to resistant disease, relapse, or early death due to advanced disease at presentation. Five out of six patients (83%) had the disease in remission, while one patient (17%) expired during induction chemotherapy before remission (Table 1).

Various factors, like age, gender, type of malignancy, and the status of diagnosis of DS before presentation, were analysed in terms of the outcome of the study cohort, but the status of diagnosis of DS before presentation was the only statistically significant factor found to be associated with the outcome (*p*-value = 0.004).

## Discussion

The first case of DS-associated acute leukaemia was published in 1930 [18]. DS is now considered the most common syndrome of leukaemia predisposition, and DS-leukaemia has exceptional clinical features with noteworthy differences in prognosis and treatment toxicity [19, 20]. There have been particular immunophenotypic characteristics of DS-ALL in the literature, with almost all patients exhibiting B-cell phenotype and rarely with T-cell or mature B-cell (Burkitt’s leukaemia) phenotype [21–23]. The same pattern of immunophenotype in DS-ALL cases has been observed in this study cohort. This study highlights the high incidence of life-threatening treatment-related toxicities in DS-acute leukaemia patients [4, 20]. The peak age of DS-ALL was also found to be the same [24], but the peak age at diagnosis of DS-AML in this study differed from the published literature [25, 26].

We also found a slightly lesser proportion of DS-associated acute leukaemia among all patients of paediatric acute leukaemia as compared to the data from HICs [27]. The ratio of B-ALL to AML in children with DS was found to be much higher (3.17) in this study as compared to the ratio (1.7) reported in previous studies from high-income countries (HICs) [3, 28]. The mean overall survival of children with DS-ALL was found to be lower (35.5 months) than children with DS-AML (42.8 months), which consolidates the findings that the overall survival rate in DS-ALL is worse than DS-AML [29].

The reasons behind the few differences observed in children with DS-acute leukaemia in this study could be the different demographical characteristics, like genetics, social and environmental factors, or it could be due to discrepancies in the data. The major limitations in this study are the retrospective study design, lack of electronic data available in our setting and small sample size. A cancer registry database is not present in our country and all of the patients’ records are kept manually in individual centres. Therefore, the data recorded retrospectively in this study might be incomplete and inadequate. Moreover, small sample size and the short study duration might not extract significant data for conclusions. But the strength of this study lies in the fact that this is the first study conducted in DS-associated paediatric acute leukaemia patients in Pakistan, and despite the innate hurdles of collection of retrospective data in a resource-limited setting, an effort has been made to publish the available data. This study calls for further prospective studies to be conducted in this special group of patients in Pakistan to draw significant conclusions, gain a better understanding of the clinical manifestations and the challenges in the management of children of DS with acute leukaemia and improve the prognosis of these patients in this part of the world too.

## Conclusion

When compared to the data from HICs, the notable differences seen in childhood acute leukaemia with DS in our setting show a lesser proportion of DS-associated childhood acute leukaemia among all cases of paediatric acute leukaemia, an older peak age of DS-AML at diagnosis and a higher ratio of DS-ALL to DS-AML. Treatment-related mortality was the sole cause of treatment failure in children of DS with acute leukaemia in our setting. Further prospective studies should be conducted on children with DS-associated acute leukaemia in developing countries.

## List of abbreviations

ALL: Acute lymphoblastic leukaemia
AML: Acute myeloid leukaemia
DS: Down syndrome

## Conflicts of interest

The authors have no conflicts of interest to disclose.

## Funding

The authors declare that they have not received any funding for this research/article.

## Figures and Tables

**Table 1.** Mortality analysis of patients with Down syndrome with acute leukaemia.

No.	Age	Gender	Type of Leukaemia	Survival (months)	Phase of therapy	Cause of death
1.	2 y	Female	B-ALL	1	Induction	Bacterial sepsis
2.	1 y	Male	B-ALL	2	Consolidation	Bacterial sepsis
3.	9 y	Male	B-ALL	17	Maintenance	Bacterial sepsis
4.	1.3 y	Female	B-ALL	5	Delayed intensification	Bacterial sepsis
5.	1.5 y	Male	B-ALL	5	Delayed intensification	Bacterial sepsis
6.	2 y	Female	AML	2	Intensification	Invasive fungal infection
